# University students dataset related to achievement, classroom practices, perceptions and attitudes of multimedia-based learning quantum physics

**DOI:** 10.12688/f1000research.128013.1

**Published:** 2023-01-03

**Authors:** Pascasie Nyirahabimana, Evariste Minani, Mathias Nduwingoma, Imelda Kimeza

**Affiliations:** 1 African Center of Excellence for Innovative Teaching and Learning Mathematics and Science (ACEITLMS), University of Rwanda College of Education (UR-CE), Kayonza, Rwanda; 2 School of Education, University of Rwanda College of Education, Kayonza, PO BOX 55 Rwamagana, Rwanda; 3 School of Education, Mbarara University of Science and Technology, Mbarara, P.O BOX 1410, Mbarara, Uganda

**Keywords:** Dataset, Conceptual understanding, Rwanda, Student academic achievement, Student attitude, Quantum mechanics, Quantum physics, University students.

## Abstract

This dataset presents data collected to assess teaching and learning of quantum physics at the University of Rwanda - College of Education (UR-CE), Rwanda. Data were collected between August and November 2019 as the baseline, and between January and April 2022 under a quasi-experimental design. Three sets of data were collected. The first set was about students’ performance and conceptual understanding collected before and after teaching intervention (lecture method or multimedia-aided approach) using mainly Quantum Physics Conceptual Survey (QPCS). The second set documented classroom practices during teaching and learning using the Classroom Observation Protocol for Undergraduate STEM (COPUS). The last set is comprised of the data related to lecturers’ and students’ perceptions before teaching and learning quantum physics and students’ attitudes after learning Quantum physics. The Quantum Physics Attitude Test (QPAT) was mainly used to collect these data. The dataset is important to education stakeholders because university managers can visualize the status of teaching and learning outcomes, lecturers can reflect on the study, and researchers can use the data to analyze various independent variables.

## Introduction

The education system in Rwanda has seen many transitions, including after the Tutsi genocide of 1994. In 2008, Rwanda transitioned from French to English as a language of instruction at all levels of education ^1^; in 2012, a policy for 12 years of basic education was adopted, ^2^ and in 2013 the public higher education institutions were merged into a single University of Rwanda. ^3^ Several studies have so far documented learning of physics ^4^^–^^6^ after a competence-based curriculum was implemented in secondary schools in 2016. ^7^ Few people have chosen the direction of higher learning institutions. ^8^ However, few of them focused on teaching and learning practices of quantum physics at the university level. Quantum mechanics is a discipline that is introduced by quantum physics, and quantum physics is a set of principles used to explain the behavior of matter and energy. Thus, these two terms were used interchangeably in this manuscript and served as a focus of data collection. This branch of physics consists of many important phenomena in nature despite the difficulty faced by students to interpret various concepts ^9^^–^^11^ such as blackbody radiation, the photoelectric effect, Compton Effect, the wave aspects of particles, De Broglie’s hypothesis, diffraction and interferences, the model of the atom, uncertainty relations, the wave function, and Schrodinger Equations. Therefore, this served as a motivation for researchers to investigate the teaching and learning of quantum physics at the University of Rwanda – College of Education (UR-CE). Before the implementation of the intervention, a baseline study was conducted. This baseline study aimed to determine the prime indicators of current teaching methodologies and students’ perceptions of quantum physics for quality knowledge delivery at the University of Rwanda College Of Education. ^9^ For this study, attitude tests (Quantum Physics Attitude Test, QPAT) for students and Educators were used. These research instruments were designed and validated by university experts in science education. We piloted them on 30 physics students and two Educators from the University of Kibungo, Rwanda. These participants were not part of this study.

## Methods

### Research design and method of data collection

The study was quasi-experimentally designed. ^12^ Before data collection was initiated, ethical clearance was applied to and approved and provided by the Unit of Research and Innovation, University of Rwanda-College of Education. Lecturers were briefed on required teaching approaches, and students and physics lecturers were informed of voluntary participation. The study was conducted at UR-CE in the department of Mathematics, Science, and Physical Education (MSPE) with groups of second-year undergraduate students from Mathematics-Physics-Education (MPE), Physics-Chemistry-Education (PCE) and Physics-Geography-Education (PGE) combinations assigned to experimental and control groups. Teaching intervention was delivered from 28 January to 1 April 2022. Lecturers in Quantum Physics were agreed to help the respective groups to understand the subject. Thus, while traditional lecture group or lecture class were taught using marker and whiteboard and PowerPoint presentations, the experimental group or multimedia class was taught using animations, PhET simulations, and YouTube videos. Animation introduced a concept while teaching with multimedia to engage students. Before playing a simulation or a video, students were given prediction questions to help them predict the outcomes of the experiment. Before intervention (December 2021), a pre-test was administered using an adapted Quantum Physics Conceptual Survey (QPCS). ^13^ It was adapted because it was not used alone, as it lacks some elements taught in the UR-CE Quantum Mechanics module. Researchers added some questions (see Table 1) about blackbody radiation from textbooks and total energy, and the probability of finding a particle from the Quantum Mechanics Conceptual Survey (QMCS). ^14^ This decision was advice from four university physics lecturers who validated the test’s content.

**Table 1.** Arrangement of achievement test items.

Total number	Numbering in the test	Correct answers	Sources
1	**Q1**	C	Questions from textbooks
2	**Q2**	A	
3	**Q3**	B	
4	**Q4**	B	
5	**Q1**	A	Questions from QPCS
6	**Q2**	B	
7	**Q3**	D	
8	**Q4**	D	
9	**Q5**	D	
10	**Q6**	A	
11	**Q7**	A	
12	**Q8**	B	
13	**Q9**	A	
14	**Q10**	C	
15	**Q11**	C	
16	**Q12**	A	
17	**Q13**	B	
18	**Q14**	C	
19	**Q15**	C	
20	**Q16**	B	
21	**Q17**	C	
22	**Q18**	A	
23	**Q19**	A	
24	**Q20**	D	
25	**Q21**	D	
26	**Q22**	A	
27	**Q23**	D	
28	**Q24**	B	
29	**Q1**	B	Questions from QMCS
30	**Q2**	B	
31	**Q3**	C	
32	**Q4**	D	

We used a test-retest method for checking reliability and the internal consistency revealed a high Cronbach alpha coefficient (a = 0.718) of 32 QPCS items used. The first author and her trained assistants made follow-ups by doing classroom observations to see whether there were any challenges during the intervention. After six weeks of teaching, the post-test was also given to all participants to examine if the multimedia intervention had some effects on students’ conceptual understanding. We used the Classroom Observation Protocol for Undergraduate STEM (COPUS) developed by Northern American researchers. ^15^ This protocol comprises 12 codes for instructor activities, 13 codes for student activities, and three codes for student engagement. Before data collection, interrater reliability were obtained between each of the two pairs of classroom observers. Student codes generated a Kappa value of 0.652, instructor codes 0.805, and student participation codes generated 0.625. These values are considered very high in inter-observer reliability. ^16^

The Quantum Physics Attitude Test (QPAT) was adapted from the Evaluation Questionnaire for Computer Simulations (EQCS) ^17^ and designed based on the literature review, daily teaching experiences, and students’ group discussions. The formulated 29 questions were content validated by five university experts in research, and the final QPAT comprised 24 items. The test had four sections: (i) College students’ perceptions of quantum physics concepts, (ii) Use of multimedia in teaching and learning quantum physics, (iii) Students’ feelings towards learning quantum physics, and (iv) the extent students recommend the actions to be taken in improving teaching and learning quantum physics. The test was implemented after getting a 0.74 reliability of internal consistency. We also need to note that while achievement data were collected before and after the intervention, classroom observation data were collected during the teaching and learning period; however, students’ attitude data were collected after teaching and learning follow-up, and the staff and students’ perceptions before teaching and learning activity.

## Description of dataset

This dataset comprises five Excel files and five pdf files. These pdf files are research tools used to collect data, while these Excel files are data files. The first file is students’ achievements (named: Data Related to Academic Achievement 2022). This file has four sheets. (1) Pre-test of the Control group, (2) Post-test of the Control group, (3) Pre-test of the Treatment group, and (4) Post-test of the Treatment group. In the sheet, the first column shows the study combination as mentioned in the above section and the second column consists of serial number (s/n). From the third column or column-C to column-HA, the students’ answer choices for each of the 32 questions are entered. Correct answers are presented above each question. From column-AK to column-PB, each question is marked. The same analysis as that in Ref. 18 was adopted. The formula used was “= IF (EXACT(E18, E$15),1,0). This means we matched the answer of the first students on row-18 and column-C with the expected answer on row-15 and column-C on the first question. The dollar sign was used to avoid the formula to shift the rows down. If the answers matched, the software assigned one mark, and a zero otherwise. We summed these scores up in column-BR and computed their respective percentage in column-BS. A total of 385 undergraduate students participated in the pre-and post-tests.

Below the data, the number of students who chose a certain answer was computed (see row-198 and column-C) using the “COUNTIF” function. After this, a percentage was computed. Likewise, the sum and corresponding percentage were calculated under the assigned marks (see row-199 and column-AK).

The second data file (named “Data related to baseline study_Post teaching and learning Quantum Physics_2022”) presents the students’ and educators’ perceptions of teaching methodologies used while teaching quantum physics. It contains two sheets with quantitative and qualitative data collected from students who have previously learned quantum physics and educators who taught this course in past years. Thus, this was used for the baseline study, where we investigated the impact of current teaching methodologies and teaching staff and students’ perceptions of quantum physics for a quality knowledge delivery system. ^9^

The third and four files are related to classroom observation data. One comprises data for the control group (named: Data related to Classroom Observation 2022_Control group), while the other comprises data for the experimental group (Data related to Classroom Observation 2022_Experimental group). These files have four sheets each. The first sheet contains raw data, the second sheet presents the meaning of codes used in the first sheet, the third sheet present analyzed data by activity, and the fourth sheet present analyzed data by time interval. Data were entered in a way that when an activity was observed, a mark of “1” was entered and when an activity was not observed, an empty space is left. The intervention was carried out for 80 minutes per week for weeks. For this reason, the first column (or column A) consists of two-minute time intervals of observed activities, and column AF shows the week (weeks 1-6). Rows 14 and 15 show the codes or observed activities, while the sum and total of observed activities are computed at the end of the table of activities. These sums are used to plot the activities graph (see sheet 3) as they do not consider time intervals. Thus, the proposition of each activity is calculated based on all activities. That is why all activities’ occurrence add up to 100. On the other hand, when considering activities and the sometimes time interval, the computed proportions of activities do not add up to 100. They can go beyond (see sheet 4). This was computed referring to the total intervals calculated in column AE. A Classroom Observation Protocol for Undergraduate STEM (COPUS) tools was used to collect and analyse data. ^15^

The fourth file data file (named “Data Related to Students Attitude_Post assessement 2022”) presents the students’ perceptions on multimedia usage in teaching and learning quantum physics attitude. It contains five sheets. The first sheet explains the codes or assigned numbers used in the second and fourth sheets. The second sheet (Experimental group _QPAT) and the third sheet (Experimental_G_QPAT-Open Q) contain quantitative and qualitative data, respectively. Likewise, the fourth sheet (Control group_QPAT) and the fifth sheet (Control_G_QPAT_Open Q) contain quantitative and qualitative data, respectively. This was done to ease the visualization of data since the attitude test contained both closed and open questions. Thus, quantitative data from closed-question items were put in a separate sheet, and qualitative data from open-ended question items were put in their separate sheets. 385 undergraduate students who have completed the learning of quantum physics using either lecture or multimedia application methods participated in the survey. The reason why fewer students attended the attitude test than those who attended the achievement test may be that the achievement test was performed on paper while the attitude test was performed online. The questions under the “Student feelings towards learning Quantum physics” were answered using five scales. Strongly agree (5), Agree (4), Neutral (3), Disagree (2), and Strongly disagree (1). Therefore to analyze such data, a COUNTIF function was again employed as in the achievement test.

## Data Availability

The data are available to use from the Mendeley repository. Readers are able to view the raw data, replicate the study, and re-analyze and/or reuse the data (with appropriate attribution). Mendeley Data: Dataset from the University of Rwanda College of Education during Learning Quantum Physics. https://data.mendeley.com/datasets/gm49fmx86t/5. ^19^ This dataset contains the following underlying data:
• Data file 1. (Data Related to Academic Achievement 2022) • Data file 2. (Data related to baseline study_Postteaching and learning Quantum Physics_2022) • Data file 3. (Data related to Classroom Observation 2022_Control group) • Data file 4. (Data related to Classroom Observation 2022_Experimental group) • Data file 5. (Data Related to Students Attitude_Post assessment 2022) Data are available under the terms of the Creative Commons Zero “No rights reserved” data waiver (CC0 1.0 Public domain dedication). Mendeley Data: Dataset from the University of Rwanda College of Education during Learning Quantum Physics. https://data.mendeley.com/datasets/gm49fmx86t/5. ^19^ There are five other files connected to these data. The achievement and attitudes tests were used to collect students’ data. The first one is named “Final Quantum Physics Conceptual Survey (QPCS)_adapted&used,” while another is named “Quantum Physics Attitude Test (QPAT)_data collection Tools_2022.” The file of a questionnaire administered to lecturers and students as a baseline survey is named “Data related to baseline study on Quantum Physics teaching and learning_2022.” The last files are the “Participants sheet and consent form” and “Researh ethical Clearance approval.”
