# Supplementary material for: University students dataset related to achievement, classroom practices, perceptions and attitudes of multimedia-based learning quantum physics
Source: F1000Res. 2024 Feb 12;12:4. Originally published 2023 Jan 3. [Version 2] doi: 10.12688/f1000research.128013.2 (PMC11009567; doi:10.12688/f1000research.128013.2)
Supplement: Peer review history

# Reviewer report: Reviewer response for version 2

**DOI:** 10.5256/f1000research.162481.r260496

**Published:** 2024-04-11

**Authors:** Wade Naylor

**Affiliations:** 1 University of Johannesburg, Auckland Park, Gauteng, South Africa; 2 National School of Education, Faculty of Education and Arts, Australian Catholic University, Brisbane, Australia

This data note uses four different inventories at the University of Rwanda - College of Education (UR-CE) in 2022: the Quantum Physics Conceptual Survey (QPCS); the Classroom Observation Protocol for Undergraduate STEM (COPUS) and the Quantum Physics Attitude Test (QPAT). The datasets are useful as faculty board / management can see the status of teaching and learning outcomes, lecturers can also reflect on their andragogy and researchers can use the data to compare with other institutions.

 One of the strengths of the data note is that it contains some interesting data which is of use to the PER community in relation to quantum mechanics (QM) andragogy, particularly in the African context. There are quite a few data spreadsheets to work through but all relevant data appears to be present and relatively clean.

 Another strength is the honest way in which the data has been presented such that other researchers can use this data to compare with their own cohorts and can hence lead to new research projects and collaborations.

 One minor weakness is the way the files are labelled and explained. This could be clearer, because as far as I can tell, there are four instruments (tests / surveys), with various Excel data files and test / survey PDFs.

 To overcome this minor weakness, I recommend that the authors label each test / survey instrument as I1, I2, I3 and I4 according to something like:

 I1_Quantum Physics Conceptual Survey (QPCS)_adapted&used_2022 / Data file 1

 I2_Quantum Physics Attitude Test (QPAT)_data collection Tools_2022 / Data file 5 -> Data file 2

 I3_COPUS Instrument - Data files 3 and 4.  -> For example renaming to Data file 3a and 3b or similar would be clearer.

 I4_Questionnaire for lecturers on the teaching of Quantum Physics_2022 / Data file 2*. -> Data file 4

 *Please also note there is a typo in the filename of I4: Questionnaire for lecturers on the teaching of Qunatum Physics_2022 -> Questionnaire for lecturers on the teaching of Quantum Physics_2022.

 For example, if the information on the inventories were placed into a Table [Refer files section],then the Data Note would be clearer making it easier for other researchers to use.

 As another referee has also pointed out, it would be interesting to have some small mention of the possible investigations the authors envisage to carry out with the data collected for the future.

 For example, in Instrument 1, the analysis of the data uses a quasi-experimental study, where the baseline data for the QPCS contain pre and post-test results and could be used to look at normalised gains. With ~180 students in each of the data sets there should be enough paired data. From Data file 1 it is not clear if the pre and post-test data in the Excel spreadsheet has the function to find students who did both the pre and post-test (paired). This is possibly beyond the scope of this data note; however, such gains (see Hake, 1998 [Ref 1]) for each cohort could be added to the spreadsheet (or as future work) if student tracking of pre to post-test was included or investigated.

 As another example, for Instrument 2, the modified Quantum Physics Attitude Test (QPAT) comprised of 24 items and it would be interesting to do some kind of factor analysis relating the QPAT to the QPCS to see how students performed between the control and experimental groups and / or pre to post-test phase. This might also be possible to do with instruments 3 and 4 as well.

 In summary, the authors have developed four instruments that were used on students at the University of Rwanda - College of Education (UR-CE) in 2022. Each instrument investigates how students and staff deal with teaching, learning and self-efficacy of quantum mechanics courses. The size of the cohorts involved are of the order of 100 and hence there are ample opportunities to do further research with this data.

 In terms of other future work, besides those mentioned above, if the paired data for instrument 1 (QPCS) are not that significant in number then another approach would be to look at dominant misconceptions (Martin-Blas T, et. al. 2010 [Ref 2]) as has recently been done in South Africa (Chrysostomou A, et al., 2024 [Ref 3]). Finally, given that there has been a switch from French to English over the last 15 years in Rwanda, it would be interesting to see how indigenous / first language correlates with the QPCS which is written in English.

Are sufficient details of methods and materials provided to allow replication by others?

Yes

Is the rationale for creating the dataset(s) clearly described?

Yes

Are the datasets clearly presented in a useable and accessible format?

Yes

Are the protocols appropriate and is the work technically sound?

Yes

Reviewer Expertise:

Physics education research, applied statistics, STEM education.

I confirm that I have read this submission and believe that I have an appropriate level of expertise to confirm that it is of an acceptable scientific standard.

## References

[1] : Interactive-engagement versus traditional methods: A six-thousand-student survey of mechanics test data for introductory physics courses. *American Journal of Physics* .1998;66(1) : 10.1119/1.18809 64-74 10.1119/1.18809

[2] : Enhancing Force Concept Inventory diagnostics to identify dominant misconceptions in first-year engineering physics. *European Journal of Engineering Education* .2010;35(6) : 10.1080/03043797.2010.497552 597-606 10.1080/03043797.2010.497552

[3] : A three-year comparative study of dominant misconceptions among first-year physics students at a South African university. *Physics Education* .2024;59(1) : 10.1088/1361-6552/ad14ea 10.1088/1361-6552/ad14ea

# Reviewer report: Reviewer response for version 2

**DOI:** 10.5256/f1000research.162481.r246076

**Published:** 2024-02-20

**Authors:** Nicolas Labrosse

**Affiliations:** 1 University of Glasgow, Glasgow, Scotland, UK

No further comment

Are sufficient details of methods and materials provided to allow replication by others?

Yes

Is the rationale for creating the dataset(s) clearly described?

Yes

Are the datasets clearly presented in a useable and accessible format?

Yes

Are the protocols appropriate and is the work technically sound?

Yes

Reviewer Expertise:

Physics Education Research; Scholarship of Learning and Teaching

I confirm that I have read this submission and believe that I have an appropriate level of expertise to confirm that it is of an acceptable scientific standard.

# Reviewer report: Reviewer response for version 1

**DOI:** 10.5256/f1000research.140564.r214701

**Published:** 2023-11-08

**Authors:** Nicolas Labrosse

**Affiliations:** 1 University of Glasgow, Glasgow, Scotland, UK

This data note provides information necessary to review and analyse the data collected by the authors. The method of data collection is well described. 

 A few more details on the teaching interventions and their pedagogical motivations would be really interesting to have. However this may be beyond the scope of this data note. 

 It would be interesting to see what possible investigations the authors envisage to carry out with the data collected.

Are sufficient details of methods and materials provided to allow replication by others?

Yes

Is the rationale for creating the dataset(s) clearly described?

Yes

Are the datasets clearly presented in a useable and accessible format?

Yes

Are the protocols appropriate and is the work technically sound?

Yes

Reviewer Expertise:

Physics Education Research; Scholarship of Learning and Teaching

I confirm that I have read this submission and believe that I have an appropriate level of expertise to confirm that it is of an acceptable scientific standard.

# Reviewer report: Reviewer response for version 1

**DOI:** 10.5256/f1000research.140564.r158997

**Published:** 2023-01-18

**Authors:** Peter C. Samuels

**Affiliations:** 1 Business School, Birmingham City University, Birmingham, UK

The datasets are definitely useful, and much effort has gone into organising them. The rationale is clear in the introduction. The method is also clearly presented. The description of the datasets is clear and helpful.

 However, there are a few issues I would recommend improving:

1. The 1994 Rwandan genocide is not known outside of Rwanda as the Tutsi Genocide. This name might cause confusion to an international audience.
2. The test-retest method for checking the reliability of the QPCS may need additional thought. Cronbach's alpha values tend to be high for scales involving larger numbers of items. The sample size is not stated. A citation should be given to justify this analysis.
3. A sample size should also be provided for the interrater reliability of COPUS.
4. Some of the data sets (such as Data related to baseline study _Post teaching and learning Quantum Physics_2022.xlsx) include very long question fields (such as Q3) and multiple answer fields. These will be difficult to analyse in the current format. Shorter names and a coding system could be used to make them more accessible.

Are sufficient details of methods and materials provided to allow replication by others?

Yes

Is the rationale for creating the dataset(s) clearly described?

Yes

Are the datasets clearly presented in a useable and accessible format?

Partly

Are the protocols appropriate and is the work technically sound?

Partly

Reviewer Expertise:

Applied statistics (scale reliability analysis), Excel, science education

I confirm that I have read this submission and believe that I have an appropriate level of expertise to confirm that it is of an acceptable scientific standard, however I have significant reservations, as outlined above.
